# Phylogeny-Guided Selection of Priority Groups for Venom Bioprospecting: Harvesting Toxin Sequences in Tarantulas as a Case Study

**DOI:** 10.3390/toxins11090488

**Published:** 2019-08-25

**Authors:** Tim Lüddecke, Andreas Vilcinskas, Sarah Lemke

**Affiliations:** 1 Department of Bioresources, Fraunhofer Institute for Molecular Biology and Applied Ecology, Winchesterstr. 2, 35394 Gießen, Germany; 2 Institute for Insect Biotechnology, Justus-Liebig-University of Gießen, Heinrich-Buff-Ring 26-32, 35392 Gießen, Germany; 3 LOEWE Centre for Translational Biodiversity Genomics (LOEWE-TBG), Senckenberganlage 25, 60325 Frankfurt, Germany

**Keywords:** spiders, Theraphosidae, phylogenetics, venomics, bioprospecting, taxonomic bias

## Abstract

Animal venoms are promising sources of novel drug leads, but their translational potential is hampered by the low success rate of earlier biodiscovery programs, in part reflecting the narrow selection of targets for investigation. To increase the number of lead candidates, here we discuss a phylogeny-guided approach for the rational selection of venomous taxa, using tarantulas (family Theraphosidae) as a case study. We found that previous biodiscovery programs have prioritized the three subfamilies Ornithoctoninae, Selenocosmiinae, and Theraphosinae, which provide almost all of the toxin sequences currently available in public databases. The remaining subfamilies are poorly represented, if at all. These overlooked subfamilies include several that form entire clades of the theraphosid life tree, such as the subfamilies Eumenophorinae, Harpactirinae, and Stromatopelminae, indicating that biodiversity space has not been covered effectively for venom biodiscovery in Theraphosidae. Focusing on these underrepresented taxa will increase the likelihood that promising candidates with novel structures and mechanisms of action can be identified in future bioprospecting programs.

## 1. Introduction

Nature abounds with bioactive molecules synthesized by species that interact with each other, either competitively or cooperatively. These species have evolved the ability to produce chemical components that increase their fitness and favor their survival, for example by antagonizing competitors, predators, prey, and pathogens or by attracting symbionts and commensals. In the search for drugs against infectious and acquired diseases, humans have often turned to such natural bioactive molecules because they have acquired outstanding pharmacologies through millions of years of subsequent evolutive optimization towards potent bioactivity for their natural function. Accordingly, many of our current drugs are natural chemical entities or their derivatives [1].

Bioactive molecules are often sourced from microbes and plants, but attention has turned more recently to animal venoms. These evolved for hunting prey, defense against predation, and intraspecific competition [2]. The in-depth survey of venoms and their components has already led to the development of several important drugs, such as the analgesic ziconotide from the cone snail *Conus magus*, the antidiabetic exenatide, a synthetic derivative of exendin-4 from venom of the beaded-lizard *Heloderma suspectum*, and the antihypertensive captopril from the lancehead viper *Bothrops jararaca* [3]. That said, all venom-derived drugs have been isolated from a small and unrepresentative minority of venomous species, in particular from the largest and most dangerous taxa. However, venom evolved convergently in metazoans multiple times [2]. Several of the lineages that successfully evolved venom systems are additionally quite diverse on the species level with fish, insects, or arachnids being some examples. Interestingly, most representatives of these groups have not yet been studied for their venom in more detail. This means that the vast majority of venomous species remain virtually unexploited [4].

**Figure 1:**
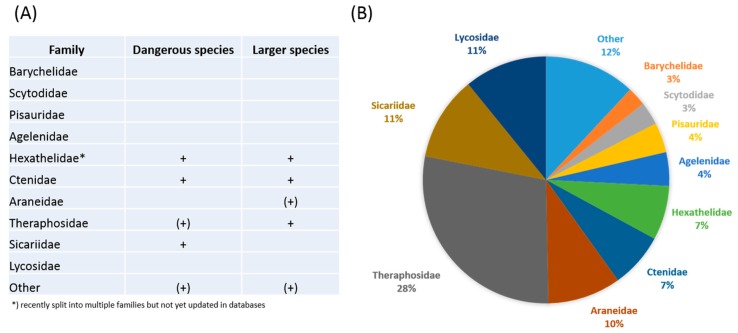
Taxonomic bias in spider venom research. Given in (**A**) are spider families which have been studied for their venom so far, together with an assignment of threat potential and size. Brackets indicate that only a small fraction of included species are either dangerous or large. Further note that the grouping “other” in reality represents the remaining 109 spider families, thus “other” contains the majority of spider biodiversity. (**B**) Visualizes the percentage of deposited toxin sequences per family. Current knowledge on spider venom is mostly based on data from those larger and more dangerous lineages and therefore is taxonomically biased. Data from [5,7,8]; see [6] for an in-depth discussion on taxonomic bias in spider venom research.

Spiders (order Araneae) provide an informative example of the problem discussed above. There are currently 48,249 recognized species of spiders, almost all of which produce venom [5], but the overwhelming majority of species that have been investigated in the search for venom-derived drugs are again either the larger or more dangerous members (Figure 1), especially from the genera *Atrax*, *Hadronyche*, *Missulena*, *Sicarius*, *Latrodectus*, *Hexophtalma*, and *Phoneutria*. This phenomenon has been coined as “taxonomic bias” and was subject to critical discussion in the recent past [6].

For biodiversity, spiders are the most successful lineage of venomous animals. The exploitation of all spiders could yield about 10 million different proteinaceous venom components, yet we have only just begun to tap this resource, with only 0.02% of such components identified thus far [8,9]. Spider venom is a promising target for bioprospecting because it is largely composed of small peptides that exhibit very specific and potent bioactivity against neuronal targets and further share an inhibitor cystine knot (ICK or knottin) motif. This structure confers a remarkable level of resistance against heat, chemicals, and proteases by structural stabilization via cysteine cross-linking, so drug candidates derived from these peptides are likely to be extremely stable in vivo [10]. Given that stability in vivo and target specificity are major constraints for suitable drug candidates, facing the sheer diversity of peptides in spider venom, it is clear that it likely harbors several yet to be discovered biologics that will almost certainly serve as drug leads in the future.

## 2. Novel Strategies in Venom Bioprospecting

The translation of natural molecules to market-ready drugs is time consuming and expensive because most drug candidates fail, and the later this failure occurs in the development pipeline the greater the cost. The risk-averse pharmaceutical industry has largely abandoned such bioprospecting studies, and the burden now falls on research organizations, which work under tight financial constraints. This issue could potentially be addressed by optimizing bioprospecting strategies, for example by introducing a rational approach for the selection of taxa for investigation. Currently, bioprospecting is biased towards species that are considered to be medically significant or according to size, accessibility, and abundance [6]. This exclusion of large swathes of biodiversity stacks the odds against the discovery of promising new leads. It would be better to include neglected venomous lineages as priority groups based on rational factors, such as phylogeny, a spread-betting approach that would improve the likelihood of discovering promising candidates by attempting to cover ‘biodiversity space’. Phylogenetic distance is, besides the species ecology, among the main drivers acting on venom evolution in terms of compound diversity, and it has been suggested before that phylogenetic distance should be acknowledged by scientists who aim to gain a holistic understanding on venom compositions within taxonomic groups [11]. The conceptual basis for using phylogenetic data as a roadmap in bioprospecting is that distantly related species are likely to evolve rather different venom profiles than closely related species and, therefore, are better candidates for yielding novel biologics [11].

Therefore, researchers should include diverse genetic lineages in their investigation to maximize the likelihood of finding such novel compounds [11]. This strategy is facilitated by the increasing availability of phylogenetic trees for animal lineages [12,13,14,15], providing quantitative data that will help with the selection of target species that represent the available biodiversity.

Bioprospecting from animal venoms was predominantly performed via pharmacological screenings, in which crude venoms or isolated toxins were subjected to specialized bioassays for each respective drug-target [16]. Although this approach was successfully applied in the past to identify promising drug leads from several species of reptiles, cone snails, and larger arachnids, among others [3], it relies on the ability to obtain meaningful amounts of venom from such organisms. Thus, the pharmacology driven strategy in venom bioprospecting is somewhat restricted to animals that are either large, easy to collect/breed, or otherwise deliver high venom yields. For the vast majority of venomous animals that are quite small, rare to find in nature, or difficult to sample for their crude venom, this approach is inappropriate [17]. However, based on the recent advances in mass spectrometry and next generation sequencing, it became possible to study even those critical taxa by means of the “methodological triad” of venomics (proteomics, transcriptomics, and genomics) [4,17,18]. The increased sensitivity and depth of instruments involved in such studies combined with significant cost reductions over the last decades allows us to identify a plethora of toxin sequences from such groups, including geophilomorph centipedes, remipede crustaceans, and pseudoscorpions [19,20,21,22,23]. In order to exploit these identified sequences for pharmaceutical applications and feed them into the value chain, they need to be synthesized or recombinantly expressed prior to extensive bioactivity tests. Facing the fact that this approach has its own drawbacks and disadvantages, it is, however, currently the method-to-choose for the study of venoms from taxa where pharmacological driven surveys fail, although it is important to note that none of these approaches will probably be able to study all venoms from all taxa alone, and rather a strategy that applies both strategies in tandem might be the most fruitful.

As a direct consequence of the ~omics-based approach, which has extensively been used for the study of spider venoms in the recent past [24,25,26], several sequence databases were erected to manage the bulk of sequence data that is created by such ~omics-based studies, with the Arachnoserver and Venomzone being two examples.

## 3. Phylogeny-Guided Selection of Priority Groups for Venom Bioprospecting: Tarantulas as a Case Study

Among spiders, the family Theraphosidae (commonly known as tarantulas) has been the subject of recent detailed phylogenetic and phylogenomic studies, revealing for the first time the deep evolutionary relationships among 12 of the 14 currently accepted subfamilies within theraphosids [15,27,28,29]. Tarantulas are particularly suitable as a case study for rational selection in the context of venom bioprospecting because many tarantula-derived toxin sequences, obtained by the application of the previously explained ~omics-based venom bioprospecting strategy, are already available in protein databases [8,19]. This unique framework means that sequence and phylogenetic data can be combined to develop, test, and validate an optimized sampling strategy based on phylogenetic distance.

Accordingly, we sourced the available data from two manually curated venom databases, specifically Arachnoserver (AS) and Venomzone (VZ) for tarantula toxin sequences, as well as the World Spider Catalog (WSC) and Tarantupedia taxonomic databases [5,8,30,31]. We also inferred phylogenetic relationships based on recently published studies of tarantula evolution [15,29]. These data were used to identify the subfamilies and genera of the family Theraphosidae that are currently underrepresented in terms of the quantity of deposited venom peptide sequences, and which should therefore be targeted in future bioprospecting studies.

At the time of writing, the two venom databases were not identical in terms of the number of deposited toxin sequences (450 sequences in AS, 532 in VZ), probably reflecting differences in the stringency of criteria for data deposition and topicality. However, the databases followed similar trends in terms of the distribution of toxin sequences among the 14 recognized Theraphosidae subfamilies (Table 1). Most sequences represented subfamily Ornithoctoninae (247 sequences in AS, 339 in VZ), followed by Selenocosmiinae (76 sequences in AS, 120 in VZ) and Theraphosinae (95 sequences in AS, 51 in VZ). The remaining subfamilies were scarcely represented (e.g., Eumenophorinae and Psalmopoeinae), or no toxin sequences were present at all (e.g., Poecilotheriinae and Thrigmopoeinae). This shows that tarantula research is strongly biased towards the Ornithoctoninae, Selenocosmiinae, and Theraphosinae, whereas the other subfamilies are left behind as a biological “black box”. The reasons for such a taxonomically-biased picture may be either the size of the respective spider, which influences the venom sampling, the availability, the ability to securely identify the spider, or a combination thereof. For an in-depth discussion about taxonomic bias in spider venom research and for solutions to the problem see [6].

**Figure 2:**
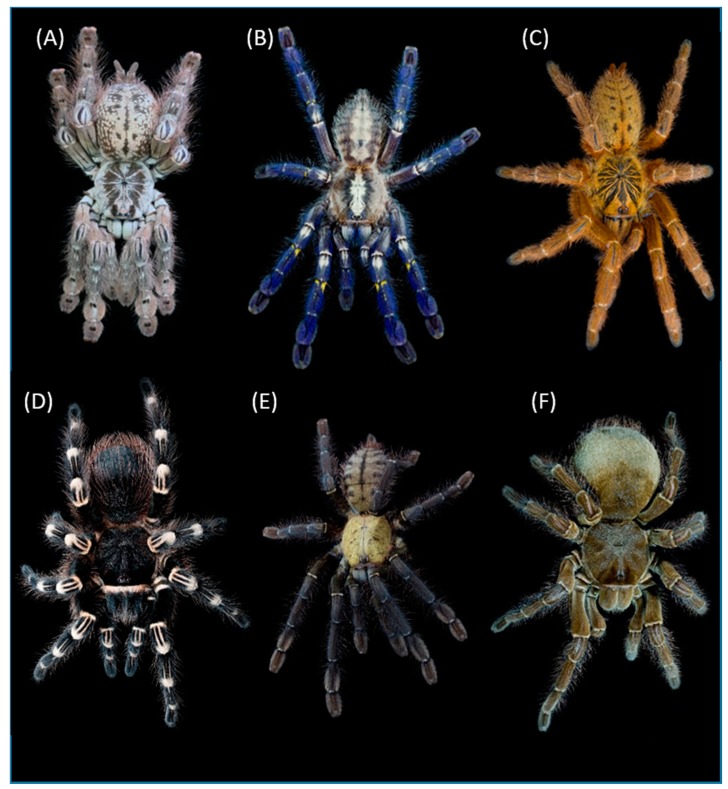
Venom-wise understudied and over-studied members of Theraphosidae. Several lineages within the theraphosid radiation were mostly neglected in the past. The upper row includes representatives of such lineages, reflecting some of the herein evaluated priority groups: (**A**) *Heteroscodra maculata* (Stromatopelminae), (**B**) *Poecilotheria metallica* (Poecilotheriinae), and (**C**) *Pterinochilus murinus* (Harpactirinae). On the other hand, some lineages are responsible for the bulk of knowledge that is available on tarantula venom: (**D**) *Acanthoscurria geniculata* (Theraphosinae), (**E**) *Cyriopagopus schioedtei* (Ornithotoctoninae), and (**F**) *Theraphosa stirmi* (again, Theraphosinae). Photography is courtesy of Bastian Rast, Switzerland.

A priori, one could hypothesize that the predominance of toxin sequences representing particular subfamilies may reflect the species richness within these taxonomic groups, but this turns out not to be the case. For example, the subfamily Ornithoctoninae was the most abundantly represented group in the venom databases, but accounts for only ~3% of tarantulas, whereas the subfamily Theraphosinae ranked third for venom but accounts for more than 50% of all known species in tarantulas [31]. Having discarded the hypothesis that the abundance of toxin data correlates with species richness, we considered the possibility that the most “dangerous” species have been prioritized for investigation. Although tarantulas are generally not considered dangerous to humans, anecdotal reports suggest that species from Asia and Africa deliver more intense bites and cause more painful envenoming effects compared to species from the Americas [32]. The subfamilies Ornithoctoninae and Selenocosmiinae are African and Asian groups, providing some evidence to support the focus on “dangerous” species, but if this is the case, it remains unclear why other African and Asian subfamilies, such as Thrigmopoeinae, Stromatopelminae, Eumenophorinae. and Harpactirinae, have been largely overlooked. Most pertinently, the subfamily Poecilotheriinae is the only group of tarantulas considered medically significant for humans [7], and yet this is currently one of the least represented groups in terms of toxin sequences deposited to the herein analyzed databases (Table 1) (representatives of over- and understudied tarantula groups are depicted in Figure 2). However, we are aware of the fact that especially the situation of Poecilotheriinae and the described toxin sequences from this subfamily is a difficult case, which will be necessary to be evaluated again soon: A bioprospecting study from 2017 had *Poecilotheria formosa*, a representative of Poecilotheriinae, among the studied taxa and described over 100 toxin sequences from its venom, using a proteotranscriptomic approach [33]. Unfortunately, these toxins were not yet added to any of the herein utilized databases and were omitted by us for consistency reasons.

**Figure 3:**
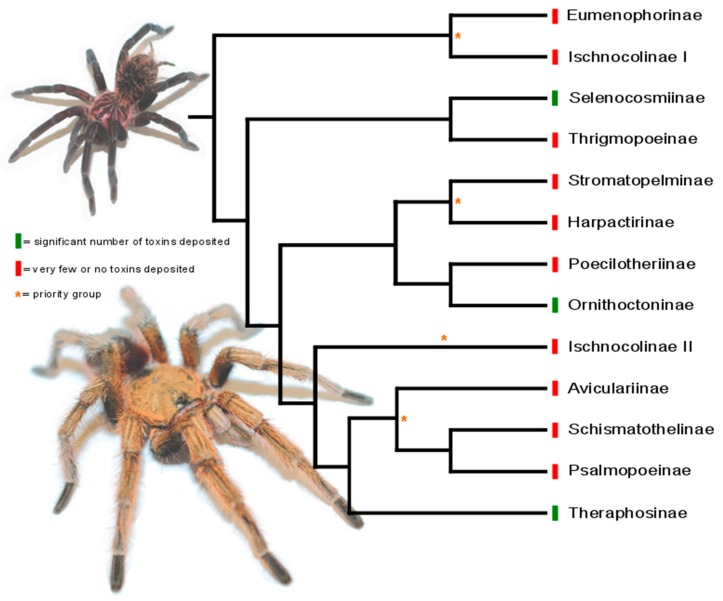
Priority groups for venom-based biodiscovery programs assigned to the tarantula tree of life, encompassing the 12 so far phylogenetically assessed subfamilies within Theraphosidae (cladogram based on [29]). The subfamilies Ornithoctoninae, Selenocosmiinae, and Theraphosinae dominate in terms of deposited toxin sequences (green), whereas little or no information is available for the other subfamilies (red). Entire theraphosid clades, representing major radiations within the family, are almost completely unrepresented and are therefore considered priority groups (red asterisk), including the clade of Eumenophorinae and Ischnocolinae, the African clade of Harpactirinae and Stromatopelminae, and the non-theraphosine new-world tarantulas. Note that the subfamily Ischnocolinae is paraphyletic and therefore appears twice in the phylogeny.

Recent evolutionary analysis on 12 out of the total 14 subfamilies within Theraphosidae revealed that the so far phylogenetically determined subfamilies form five major clades, representing distinct genetic lineages (Figure 3). If the distribution of toxin data for each subfamily is mapped onto this phylogeny, it becomes clear that several of these major clades have been neglected in previous studies: This is true for the clade formed by subfamily Eumenophorinae and parts of the paraphyletic Ischnocolinae, the clade formed by the African subfamilies Harpactirinae and Stromatopelminae, and the non-theraphosine members of the clade, comprising American subfamilies Aviculariinae, Schismatothelinae, and Psalmopoeinae, plus some species of Ischnocolinae. Given the clear bias in the coverage of toxin sequences and the plethora of toxins anticipated in these three underrepresented clades, plus their evolutionary distance from other tarantula subfamilies, we propose that the members of these clades should be prioritized in future bioprospecting studies. The subfamilies Poecilotheriinae and Thrigmopoeinae are likewise underrepresented, but given their closer relationship to Ornithoctoninae and Selenocosmiinae, they are probably less likely to harbor really novel toxins.

## 4. Concluding Remarks

The advent of venom-focused genomics, transcriptomics, and proteomics has provided the means to study venoms in a high-throughput and cost-effective manner [34,35]. We are confident that the application of venomics to the priority groups we have identified will contribute to the understanding of theraphosid venoms and will help to accelerate venom-based biodiscovery programs focusing on these intriguing and charismatic spiders. Apart from the family Theraphosidae, the phylogeny-guided bioprospecting approach herein discussed might further accelerate biodiscovery from very diverse venomous lineages in general. For example, across the spider tree of life alone, multiple clades have been phylogenetically resolved recently but remain either completely unstudied for their venom so far or all available information on venoms from these clades is derived only from one or two species [8,36,37,38,39,40], thus reflecting a very narrow fraction of these lineages. Gaining a thorough understanding of the venom composition for those groups is a major challenge if a complete idea upon arachnid venoms wants to be achieved, may it be for bioprospecting or for basic research on the biology of spiders itself.

Independent of studied taxa or research aims, the study of venoms from understudied groups needs to be performed more rapidly. On one hand, this is important for the aforementioned streamlining and economization of bioprospecting programs, but on the other hand, this is of pivotal importance in order to create something akin to a “library of bioresources” from venomous animals. We are currently living in the sixth mass extinction event, and the dramatic loss of global biodiversity likely affects many toxin-producing species, as powerfully highlighted by the dramatic biodiversity loss in amphibians [41,42,43,44,45,46]. Consequently, it is a real concern that valuable bioresources which could be found in such animals are getting lost forever, in case the respective species goes extinct. Therefore, it is a major task for the toxinological community to enhance the studies of venoms and create such a “library of bioresources” in order to save the genetic information of venom proteins for the future.

The comprehensive study of venom as a bioresource suffers from a variety of problems that affect its success rate. For example, many of the unstudied venomous species are rather small, and it is notoriously difficult to obtain meaningful amounts of venom for bioactivity screens or proteomic studies from these. Furthermore, some of these species are difficult to study because of their secretive lifestyle or their natural habitat being cumbersome to explore [4,35]. Additionally, political restrictions, such as those imposed by the Nagoya Protocol, are major impediments that somewhat negatively affect venom bioprospecting (see [47] for a discussion on the topic using microbiology as an example). Beyond these major problems, which certainly need to be solved in the future, the rational selection of taxa by means of phylogenetic distance could drastically improve any research efforts in this direction and contribute to the achievement of such a goal.

## Figures and Tables

**Table 1:** Species richness in the family Theraphosidae and the number of toxin sequences deposited in the databases Arachnoserver (AS) and Venomzone (VZ) plus species diversity from World Spider Catalog (WSC) for each subfamily. Although the number of deposited sequences differs between the databases, they follow the same trend with the majority of sequences representing subfamilies Ornithoctoninae, Selenocosmiinae, and Theraphosinae. The remaining subfamilies are poorly represented, if at all. Subfamilies with unclear phylogenetic placement are marked with astersisks.

Subfamily	Number of Species WSC	Number of Toxins AS	Number of Toxins VZ
Acanthopelminae ^1^	2	0	0
Aviculariinae	31	0	1
Eumenophorinae	62	10	1
Harpactirinae	62	8	7
Ischnocolinae ^2^	85	3	0
Ornithoctoninae	27	247	339
Poecilotheriinae ^3^	14	0	0
Psalmopoeinae	27	6	7
Schismatothelinae ^2^	21	0	0
Selenocosmiinae	114	76	120
Selenogyrinae ^1^	10	0	0
Stromatopelminae	10	5	6
Theraphosinae	526	95	51
Thrigmopoeinae	9	0	0

^1^ subfamily with unclear phylogenetic placement., ^2^ paraphyletic, ^3^ toxins described but not yet added to databases.
